# Comparative morphology of the whiskers and faces of mice (*Mus musculus*) and rats (*Rattus norvegicus*)

**DOI:** 10.1242/jeb.245597

**Published:** 2023-10-12

**Authors:** Chris S. Bresee, Hayley M. Belli, Yifu Luo, Mitra J. Z. Hartmann

**Affiliations:** ^1^ Northwestern University Institute for Neuroscience, Northwestern University, Evanston, IL 60208, USA; ^2^ Department of Biomedical Engineering, Northwestern University, Evanston, IL 60208, USA; ^3^ Department of Mechanical Engineering, Northwestern University, Evanston, IL 60208, USA

**Keywords:** Synthetic data, Multimodal, Sensorimotor integration, Active sensing, Vibrissa, Whisker, Touch, Tactile, Vision, Audition, Nares, Sniffing, Neuromechanics

## Abstract

Understanding neural function requires quantification of the sensory signals that an animal's brain evolved to interpret. These signals in turn depend on the morphology and mechanics of the animal's sensory structures. Although the house mouse (*Mus musculus*) is one of the most common model species used in neuroscience, the spatial arrangement of its facial sensors has not yet been quantified. To address this gap, the present study quantifies the facial morphology of the mouse, with a particular focus on the geometry of its vibrissae (whiskers). The study develops equations that establish relationships between the three-dimensional (3D) locations of whisker basepoints, whisker geometry (arclength, curvature) and the 3D angles at which the whiskers emerge from the face. Additionally, the positions of facial sensory organs are quantified relative to bregma-lambda. Comparisons with the Norway rat (*Rattus norvegicus*) indicate that when normalized for head size, the whiskers of these two species have similar spacing density. The rostral–caudal distances between facial landmarks of the rat are a factor of ∼2.0 greater than the mouse, while the scale of bilateral distances is larger and more variable. We interpret these data to suggest that the larger size of rats compared with mice is a derived (apomorphic) trait. As rodents are increasingly important models in behavioral neuroscience, the morphological model developed here will help researchers generate naturalistic, multimodal patterns of stimulation for neurophysiological experiments and allow the generation of synthetic datasets and simulations to close the loop between brain, body and environment.

## Teaser

**Summary:** A morphological model of the whiskers and facial features of mice can be used in behavioral simulations and to generate naturalistic patterns of stimulation for neurophysiological experiments.

## INTRODUCTION

Animal nervous systems evolve simultaneously with their peripheral sensory structures. To understand the signals that an animal's brain evolved to process thus requires consideration of the relative positions and orientations of the animal's sensors. The spatial arrangement of facial sensors is particularly important to the study of multimodal integration, as this arrangement directly shapes how visual, auditory, olfactory and tactile signals are actively acquired by the animal and received by the brain.

Given that house mice (*Mus musculus*) and Norway rats (*Rattus norvegicus*) are the most common model species used in basic neuroscience and biomedical research, it is surprising that the spatial arrangement of their sensory structures has not yet been quantified and compared. For both species (‘mouse’ and ‘rat’), one of the most important sensory organs is the whisker (vibrissal) array, which provides a set of rich tactile cues during multiple behaviors, ranging from locomotion and climbing, to texture discrimination, to social interactions (Grant et al., 2018; Guić-Robles et al., 1989; Wolfe et al., 2011). The behavioral importance of the whiskers is paralleled by the hypertrophy of whisker-related structures within the rodent nervous system, particularly in the ‘barrel’ region of the primary somatosensory cortex (Ma and Woolsey, 1984; Rice et al., 1985; Van Der Loos, 1976; Woolsey and Van der Loos, 1970); the whisker system is thus also ideal for electrophysiological study.

The present study performs quantitative, morphological quantification and comparison of the facial features of mice and rats with a particular focus on the vibrissae. The motivation for the work is three-fold, as the results fill scientific and technological gaps across multiple domains of research.

One motivation is to develop a morphometric approach to compare the spatial arrangement of facial structures and whiskers across species, regardless of whether the whisker arrays are ‘ordered’ into rows and columns or ‘disordered’, with no apparent row/column arrangement (Muchlinski, 2010; Muchlinski et al., 2013). Although rats and mice happen to have similar row/column arrangements of whiskers, the present work compares their morphology without relying on this similarity.

Another motivation is that a mouse morphological model would benefit behavioral and neurophysiological research in multiple ways: (1) The model could be combined with videographic data to help quantify exploratory and sensory acquisition behaviors, as has been done already for the rat (Hobbs et al., 2015, 2016). For example, overlaying the model on videos of a behaving mouse would allow researchers to estimate how the animal's sensory structures are oriented relative to an object and how sensory signals arrive at each structure relative to each other; the latter is critical for analysis of multimodal integration. (2) Combining the model with equations of motion for the whiskers (Knutsen et al., 2008) could improve whisker tracking, which remains as yet a challenging image-processing problem. (3) Quantifying the arrangement of the whiskers is a necessary first step towards creating mechanical models to estimate the mechano-tactile input to the system, so that these input signals can be correlated with neurophysiological and behavioral recordings. (4) Quantifying the arrangement of the whiskers could aid in the design of stimuli for neurophysiological experiments. For example, a researcher could construct a stimulus to move through the array of an anesthetized animal that deflected some whiskers but not others, or that deflected the whiskers in a particular sequence or temporal pattern. (5) The morphological model could ultimately enable development of simulations to examine the mechanosensory signals generated during natural tactile exploration, enabling the development of synthetic datasets that close the loop between the animal's sensorimotor structures, the environment, and the nervous system.

A final motivation for the present work is to quantitatively compare the facial and vibrissal morphologies of the mouse and rat. As elaborated in the Discussion*,* there are several reasons to expect these morphologies to be similar, but equally good reasons to expect them to differ. The quantitative comparison performed in the present work allows us to suggest that the rat is ‘scaled up’ from its most recent common ancestor with the mouse.

To summarize, the present work quantifying the facial features of *Mus musculus* was undertaken in order to fill gaps across multiple fields. The work develops an important tool that we anticipate will be used widely in the study of morphometrics, animal behavior, multimodal integration and neurophysiology. We anticipate the model will enable the creation of synthetic datasets of sensory acquisition behaviors that help close the loop between the brain, body and environment (Bolaños et al., 2021; Hobbs et al., 2016; Ramalingasetty et al., 2021; Weiler et al., 2022 preprint; Zhuang et al., 2017; Zweifel et al., 2021)

## MATERIALS AND METHODS

The Animal Care and Use Committee of Northwestern University approved all procedures in advance.

### Data collection

A total of 467 macrovibrissae from eight mice were collected for the present work. All animals were male C57BL/6J mice between the ages of 6 and 8 weeks. Animals were housed communally (2–5 mice per cage) in standard rectangular laboratory caging on a 12 h:12 h light:dark cycle with wood chip bedding and a wire top.

Data for the rat were obtained from a previous study (Belli et al., 2018), which developed equations based on a total of 518 macrovibrissae from nine female Long Evans rats between the ages of 5 and 36 months.

A limitation of the present study is its exclusive use of male mice, whereas Belli et al. (2018) used only female rats. We expect that differences between individuals of the same species will likely be confined to size rather than the relative locations of facial structures or whisker geometry. We also expect that any potential differences between males and females of the same species and similar size will be smaller than the spatial resolution of the Microscribe™ scanning approach. Future work should use animals of both sexes to confirm these expectations.

#### Surgery and anesthesia

Animals were anesthetized with a mixture of ketamine/xylazine/acepromazine injected intraperitoneally (100 mg kg^−1^ ketamine hydrochloride, 10 mg kg^−1^ xylazine hydrochloride and 3 mg kg^−1^ acepromazine maleate in a saline vehicle). Every 15 min, the toe pinch withdrawal reflex was assessed and booster doses of anesthetic were given if needed to maintain deep anesthesia. The animal was secured with bite block and ear bars in a stereotaxic apparatus and placed on a heating pad. Next, to ensure that no vibrissae touched any surface, we performed a surgery to allow head fixation without the bite block and ear bars. An incision was made along the midline of the scalp and skull screws were inserted to form a triangle: one screw was placed in the frontal bone and one screw in each temporal bone. A bridge of methyl methacrylate (dental acrylic) was constructed between the skull screws and the arm of the stereotaxic unit. We were careful to ensure that the skull landmarks lambda and bregma remained exposed (Paxinos et al., 1985). The bite block and ear bars were removed after the acrylic cured to ensure that no object touched any vibrissa or any part of the animal's face.

Because all measurements were made in the anesthetized animal, the locations of all sensory organs including the whiskers were obtained when all muscles were relaxed. The measured angles at which the whiskers emerge from the face are thus by definition the ‘resting’ angles.

#### Microscribe™ data acquisition

The Microscribe*™* is a passively movable mechanical arm with five degrees of freedom. At the start of an experiment, a probe of known length is placed on the end of the arm and an origin (0,0,0) is defined. The user moves the probe tip to a chosen location, recorded as (*x*, *y*, *z*) coordinates relative to the origin. For these experiments, we used a custom Microscribe™ probe, consisting of a 500 μm diameter tungsten bipolar electrode held in a pin vice that had been machined to thread into the Microscribe™. We followed the manufacturer's instructions to recalibrate the custom tip before collecting data from each animal.

The experimenter held the Microscribe™ and sequentially placed the probe tip at a series of points on each of the anatomical structures of interest. The 3D coordinates (*x*, *y*, *z*) at the position of the Microscribe™ tip were recorded by pressing a trigger button. We digitized multiple facial features for seven of the eight mice, including the skull features lambda and bregma, points corresponding to the corners and contours of the nostrils, eyes, mouth, rostrum, incisors, pinnae, the basepoint of each whisker and 4–60 points along each whisker.

On average, 30 whiskers could be measured on each mouse mystacial pad, compared with the 31 whiskers measured for rats in a previous study (Belli et al., 2018). This difference is due to the small size and delicate nature of rostral mouse whiskers and does not necessarily reflect a difference in the number of macrovibrissae between the two species. Because sling muscles were not identified in the present work, it was not possible to definitively determine the transition from macro- to micro-vibrissae.

#### 2D whisker scanning

After data collection with the Microscribe™ was finished, animals were euthanized with an overdose of ketamine/xylazine/acepromazine and subsequent decapitation. Whiskers from seven of the eight mice (mice 1–7) were cut at their base, using forceps and microscissors, and stored for 1 or 2 days in folded rectangles of aluminum foil, which were then stored in plastic bags. Extreme care was taken to cut the whiskers exactly at the base, pushing carefully and delicately on the skin to ensure the full arclength of the whisker was obtained. In initial experiments, we tried plucking each whisker, but found that the top of the follicle could not be consistently identified using shape, diameter or color changes. For example, some plucked whiskers tended to pull out more cleanly from the follicle than others, increasing their apparent length by a few mm. By using microscissors to cut carefully at the skin surface, we captured the full arclength of the whisker just as it emerged from the follicle. After cutting, whiskers were scanned on a flatbed scanner (Epson Perfection 4180 Photo) at a resolution of 2400 dpi (10.6 microns per pixel), along with a ruler for calibration (1 mm resolution). Whiskers from the eighth mouse were accidentally lost.

### Definition and quantification of whisker morphological parameters

Following convention (Dörfl, 1982), approximately 30 large whiskers on each mystacial pad were taken to be macrovibrissae, including whiskers up to column 7 in rows that had whiskers in these positions. More rostral whiskers became increasingly short and fine so that visual identification and mechanical manipulation were not feasible.

Whisker array morphology was quantified using a total of eight parameters, all of which have been defined in previous work (Belli et al., 2018; Knutsen et al., 2008; Luo and Hartmann, 2023; Towal et al., 2011). The arclength (*S*) and the intrinsic curvature coefficient (*A*) describe 2D whisker geometry. The term ‘arclength’ is defined as the total, curved length of the whisker, to distinguish it from the ‘chord length’ that describes the straight line between the whisker's base and its tip. The 3D coordinates of the whisker basepoints are denoted as *r*_bp_, θ_bp_ and ϕ_bp_, and the orientation at which the whiskers emerge from the pad are described by the three angles θ_w_, ϕ_w_, ζ_w_.

#### Quantifying whisker arclength and the intrinsic curvature coefficient

The 2D images of whiskers from seven mice (mice 1–7) were imported into MATLAB™ to measure arclength (*S*) and curvature coefficient (*A*). Standard semi-automated image processing techniques were used to create a trace along the whisker length. This trace was then smoothed in the y-dimension using a 20 pixel moving average filter and whisker arclength *S* was calculated by summing the lengths of the segments between the data points. Measurement error was estimated as two pixels on each end of the whisker, or ∼10.6 μm per pixel×2 pixels per endpoint×2 endpoints=∼42.4 μm.

Previous studies have shown that the 2D shape of a rat whisker can be approximated by the parabola *y*=*Ax*^2^ (Belli et al., 2018; Knutsen et al., 2008; Towal et al., 2011). The coefficient *A* is called the ‘intrinsic curvature coefficient’, or simply ‘curvature coefficient’. As will be shown in the Results, we found that a quadratic fit was also a good approximation for whisker curvature in mice.

We determined the value for *A* following the steps described in a previous study (Belli et al., 2018). The whisker was first oriented concave up, with its basepoint at the origin (0,0) and the initial portion of the whisker aligned with the *x*-axis such that the majority of the whisker lay in the first quadrant. Because previous studies have indicated that only the proximal ∼65% of the whisker remains planar (Knutsen et al., 2008; Towal et al., 2011), the arclength of the whisker was truncated to 65%.

Although a detailed analysis of whisker intrinsic curvature lies outside the scope of the present work, a recent study reviewed various methods for its quantification (Luo and Hartmann, 2023). The study examines the whiskers of 10 different species, including *Mus musculus*, and the analysis includes the full whisker arclength, instead of limiting to the proximal 65%. For full-length *Mus musculus* whiskers, a fractional exponent fit between quadratic and cubic was found to be the most accurate (Luo and Hartmann, 2023).

An iterative optimization routine that minimized the difference between the smoothed traced whisker and the fitted curve was used to determine the fraction of the whisker that should be aligned with the *x*-axis. We tested values between 1% and 30% of the whisker length. For each iteration, the curve *y*=*Ax*^2^ was fitted to the truncated whisker and the mean squared error (MSE) between the fitted curve and whisker trace was minimized. On average across all whiskers, using 8% of the proximal whisker to align with the *x*-axis generated the smallest MSE. This percentage was therefore used for alignment for all whiskers.

#### Comparing rat and mouse base diameters

To compare the base diameters of the whiskers of mice and rats we used publicly available datasets from Hires et al. (2016) and Belli et al., 2018. The Hires dataset includes 28 whiskers: three each of the A2, B2, C1, C3, C4, D2 and E2 whiskers, and seven C2 whiskers. We selected whiskers from the Belli dataset to match those measured in Hires for a total of 146: 16 A2; 20 B2; 18 C1; 19 C2; 20 C3; 18 C4; 17 D2; and 18 E2 whiskers.

#### Choice of coordinate system

**Fig. 1.**
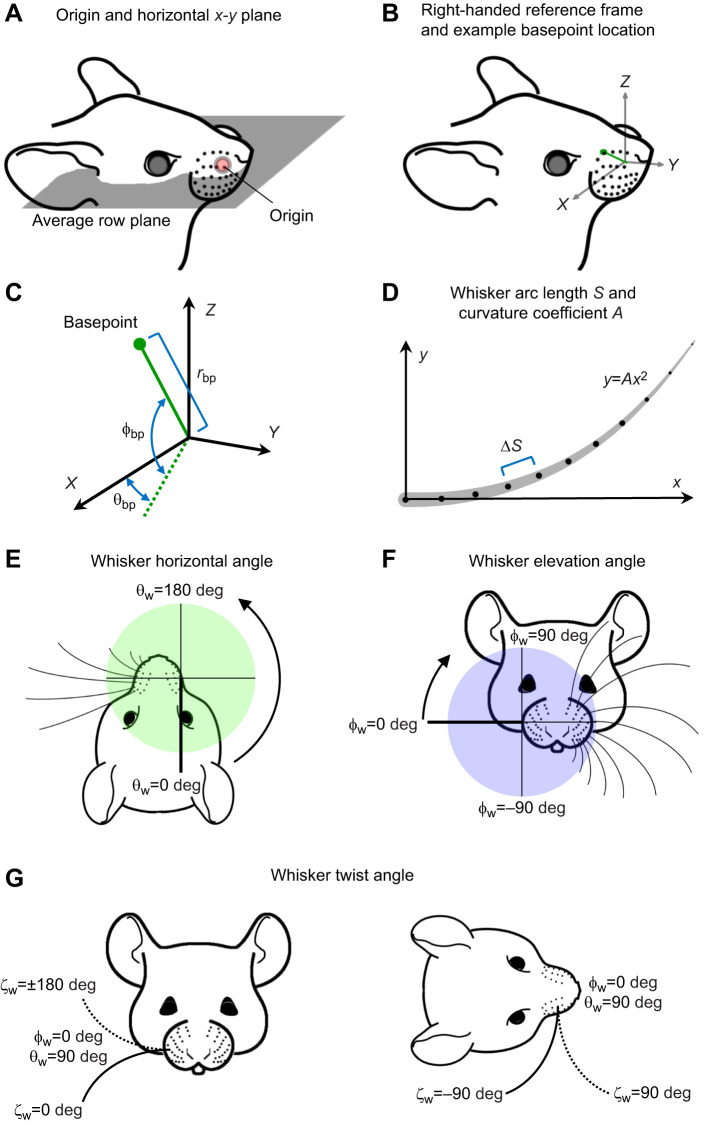
**Whisker basepoint coordinates and angles of emergence.** (A–C**)** Whisker basepoint coordinates. (A) The origin (red dot) is the average of all left and right whisker basepoint locations, and thus centered in the middle of the head (not on the face). The horizontal plane is the ‘average row plane’, defined as the mean of the planes fitted to each of the five whisker rows. (B) Axis conventions for the head. The positive *x*-axis points through the centroid of the right array, the positive *y*-axis points rostrally and the positive *z*-axis points dorsally. (C) Whisker basepoints are described in spherical coordinates as radius (r_bp_), horizontal angle (θ_bp_), and elevation angle (ϕ_bp_). (D) Whisker shape parameters. Whisker arclength (*S*) is defined as the sum of the lengths of all segments along the whisker. The whisker's intrinsic curvature coefficient (*A*) is defined by the parabola that best approximates the whisker shape in 2D. (E–G) Whisker emergence angles. (E) A top-down view of the mouse's face illustrates the coordinate system for θ_w_, the horizontal emergence angle. (F) A front view of the mouse's face illustrates the coordinate system for ϕ_w_, the elevation emergence angle. In both E and F, thin dashed lines represent the proximal, approximately linear, region of the whisker to illustrate that neither horizontal nor elevation angles of emergence depend on the intrinsic curvature of the whisker. (G) The twist angle ζ_w_ defines the orientation of the whisker about its own axis. Solid and dashed lines represent extreme positions of the whisker in the front and top views. Although ζ_w_ is formally calculated as a rotation about the *y*-axis (which points rostral–caudal), this panel illustrates ζ_w_ for the case that θ_w_=90 deg and ϕ_w_=0 deg in order to show the whisker in a more natural position. ζ_w_=0 deg points concave down, ζ_w_=180 deg concave up, ζ_w_=90 deg concave forward and ζ_w_=−90 deg concave backwards. Figure adapted from Belli et al. (2018), where it was published under the terms of a CC BY 4.0 license.

Establishing the reference frame in which to define the whisker pad parameters (*r*_bp_, θ_bp_, ϕ_bp_, θ_w_, ϕ_w_­, ζ_w_) required us first to choose an origin and a horizontal plane in which to orient the mouse's head. Following procedures previously established for the rat (Belli et al., 2018), the origin was chosen to be the mean position of all whisker basepoint locations on both left and right sides of the array. This choice placed the origin near the center of the muzzle, inside the animal's head (Fig. 1A). Note that this procedure required ‘matched’ basepoints between right and left sides, so if a particular whisker identity was present on only one side it was omitted from the calculation of the origin.

Again following the approach of previous studies (Belli et al., 2018; Towal et al., 2011), we defined the ‘average whisker row plane’ as the horizontal (*x*–*y*) plane. A plane was fitted to the basepoints of each of the five whisker rows using least squares, and the mean of these five planes defined the average whisker row plane (Fig. 1B).

With the *x*–*y* plane established as the average row plane, the *x*-axis was defined as the line connecting the centroids of the left and right arrays with the left side of the animal negative. The *y*-axis was defined to be orthogonal to the *x*-axis, with the positive *y*-axis pointing rostrally. Lastly, the axis perpendicular to the *x*–*y* plane was the *z*-axis (Fig. 1C).

#### Quantifying 3D coordinates of the whisker basepoints

Having chosen an origin and horizontal plane, the 3D coordinates of all recorded points on the animal's head, including the whisker basepoints, could be determined. We used an intuitive coordinate system that exploits bilateral symmetry of the animal by mirroring the left array across the midline. All equations and figures in the present work are defined in terms of right-sided whisker arrays. These ‘array-centered’ axis conventions apply equally well to both sides of the head, with the caveat that the coordinates on the left side of the animal no longer follow the right-hand rule.

The three spherical coordinates for basepoints (*r*_bp_, θ_bp_, ϕ_bp_) are illustrated in Fig. 1D. The radius (*r*_bp_) is defined as the straight-line distance between the basepoint and the origin and is closely related to the size of the mouse's mystacial pads. The coordinate θ_bp_ describes the rostro-caudal location of the whisker basepoint along the positive *x*-axis. If the whisker is rostral [caudal] to the *x*–*z* coronal plane, it has a positive [negative] value of θ_bp_. The dorsoventral location of the basepoints with respect to the *x*–*y* horizontal plane is described by the coordinate ϕ_bp_. If the basepoint location is dorsal to this horizontal plane it has a positive value of ϕ_bp_ whereas locations ventral to the plane have a negative ϕ_bp_.

#### Quantifying the 3D angles at which the whiskers emerge from the face

The ‘angles of emergence’ describe the orientation of the whiskers as they emerge from the mystacial pad at their basepoint locations. In the present work, the angles are defined as Euler angles applicable to the right array. The angles θ_w_ and ϕ_w_ both describe the orientation of the approximately linear portion (proximal 8%) of the whisker, near its base; neither of these angles depends on the intrinsic curvature of the whisker. As shown in Fig. 1E, the angle θ_w_ is the angle of emergence in the horizontal plane, and ranges between 0 deg (whisker points caudally) and 180 deg (whisker points rostrally). Values of θ_w_ greater than 180 deg indicate that the whisker points across the rostro-caudal midline of the animal. The angle ϕ_w_ describes the elevation of the whisker (Fig. 1F). Values range between ±90 deg, with 90 deg indicating that the whisker points dorsally and −90 deg ventrally.

Because each whisker has an intrinsic curvature, a third angle, ζ_w_, is required to describe the whisker's twist about its own axis, as illustrated in Fig. 1G. If the whisker is oriented concave forward, it has an angle of ζ_w_=90 deg, whereas concave backwards is ζ_w_=−90 deg, and concave dorsal and ventral are 180 deg and 0 deg, respectively.

As indicated earlier, the angles of emergence are defined only for whiskers of the right array. All left side whisker arrays were mirrored across the *y*–*z* plane, and figures and equations in the present work are presented in terms of right-sided whisker arrays.

To find the angles of emergence, we performed an optimization using the built-in MATLAB™ function ‘fmincon’. The optimization parameters were θ_w_, ϕ_w_, ζ_w_, *S* and *A*. Starting with the whisker point cloud in standard position and orientation, the individual points were sorted by distance from the basepoint. The *x*, *y*, *z* coordinates of all points were smoothed with a moving average filter (window size of five points), and the whisker was then resampled into 500 μm segments and its base translated to the origin. The routine next varied θ_w_, ϕ_w_­, ζ_w_, *S* and *A* to fit an idealized whisker model to the point cloud. The model whisker began with its base at the origin and its proximal portion aligned along the negative *y*-axis. The model was initially oriented concave down. To match the idealized model with the whisker point cloud, we performed an Euler rotation sequence in the order *y*-axis (roll, ζ_w_), *x*-axis (pitch, ϕ_w_), *z*-axis (yaw, θ_w_). These rotations were extrinsic about the global *y–x–z* axes. The parameters *S* and *A* were also varied to best match the idealized model to the point cloud. The optimization minimized the mean sum-squared distance between the whisker point cloud and the points in the idealized whisker model.

### Relationship between basepoint coordinates and row and column identity in the mouse

The present work quantifies the morphology of the mouse vibrissal array using the 3D position of the whisker basepoints (θ_bp_, ϕ_bp_, *r*_bp_). To do so, it was first necessary to develop equations that related the basepoint coordinates for each whisker to its row and column position. In all equations, angles are measured in degrees and lengths in mm.

As expected, θ_bp_ was linearly related to column (Col) and ϕ_bp_ was linearly related to row (Row):
(1)

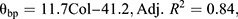

(2)

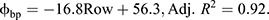


In Eqns 1 and 2, Col ranges between 1 and 7 and Row ranges between 1 (for the A row) and 5 (for the E row). Note that numbering the columns starting with 1 for the ‘Greek arc’ is nontraditional, but follows the nomenclature used in Belli et al., 2018, and accommodates comparisons with species that do not have an easily identifiable Greek arc.

**Fig. 2.**
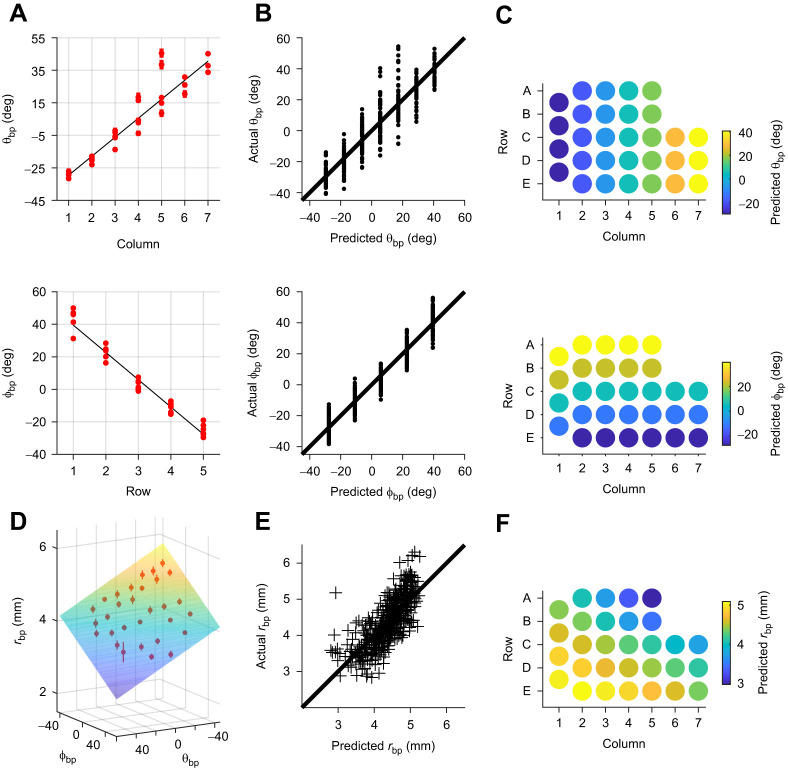
**Relationships between whisker basepoint coordinates (*r*_bp_, θ_bp_, ϕ_bp_) and row and column position in the mouse array.** (A) The basepoint coordinate θ_bp_ (*N*=447) varies linearly with column identity (Column; top panel) while ϕ_bp_ (*N*= 442) varies linearly with row identity (Row; bottom panel). Red dots show mean values when grouped by Row or Col. Black lines show Eqns 1 and 2. (B) Plots of actual versus predicted values for θ_bp_ and ϕ_bp_ highlight quality of fits for Eqns 1 and 2. (C) Predicted values for θ_bp_ and ϕ_bp_ across the array. (D) Eqn 4 represents a plane, with *r*_bp_ (*N*= 440) inversely correlated with both θ_bp_ and ϕ_bp_. Red dots and bars show mean values and s.e.m. (E) A plot of actual versus predicted values for *r*_bp_ reveals higher variability than for θ_bp_ or ϕ_bp_. (F) Predicted values for *r*_bp_ across the array.

Eqn 1 indicates that θ_bp_ increases from caudal to rostral across the mystacial pad, and Eqn 2 indicates that ϕ_bp_ decreases from dorsal to ventral; both of these relationships are shown in Fig. 2A. The quality of both fits is presented in Fig. 2B, whereas Fig. 2C visualizes the strong relationship between θ_bp_ and column, and between ϕ_bp_ and row.

A third coordinate, *r*_bp_, is needed to fully describe basepoints in spherical coordinates. We first found *r*_bp_ as a function of row and column position:
(3)

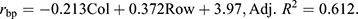


The coordinate r_bp_ is more variable than θ_bp_ or ϕ_bp_, reflected by the lower *R*^2^, which is partially due to head size differences across individual mice. We next quantified the relationship between *r*_bp_ and the basepoint parameters θ_bp_ and ϕ_bp_, and found *r*_bp_ to be linearly related to both variables (Fig. 2D):
(4)

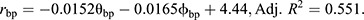


Although Eqn 4 has a slightly lower *R*^2^ value than Eqn 3, it allows comparisons between species with different numbers of rows and columns. A sense for the quality of fit of Eqn 4 is given by the dispersion about the identity line in Fig. 2E. Fig. 2F illustrates the variation of *r*_bp_ across the array, revealing the narrowing of the mouse's snout rostrally and dorsally.

### Statistical analysis of morphological parameters and error analysis

We used the method of ‘forward selection’ to incrementally increase both model order and the number of independent variables included in a model, starting with simple first-order linear regressions. We evaluated all models based on three metrics: first, the significance (as indicated by *P*<0.05) of the overall model and of individual covariates; second, the information lost by imposing a model on the dataset [as indicated by an Akaike Information Criterion (AIC) of <2 points compared with a competing model]; and third, visual assessment of good correspondence between observed and predicted values, as indicated by a qualitatively ‘tight’ clustering of points about the identity line on an observed versus predicted plot. Data for individual mice can be found at: https://doi.org/10.5281/zenodo.7992354.

#### Intrinsic curvature and arclength as functions of θ_bp_ and ϕ_bp_

A total of 357 unique 2D scanned images of whiskers were obtained from seven of the eight mice. Values of whisker arclength were obtained from all 357 scans and values of the intrinsic curvature coefficient were obtained for 324 of the scans. To identify outliers, the mean and standard deviation were calculated for whiskers grouped by their row and column identity. We eliminated whiskers greater than two standard deviations above or below the mean. Out of the dataset of 357 scans, 14 outliers (3.92%) for arclength (*S*) were removed, yielding a total of 343 whiskers. Out of the dataset of 324 scans for curvature coefficient, 12 outliers (3.70%) were eliminated, yielding a total of 312 whiskers for analysis.

We constructed models for *S* and *A* as functions of θ_bp_ and ϕ_bp_. To avoid overfitting, models were not fitted directly to the entire dataset for each parameter. Instead, the best fit model was found for each mouse individually. The best fit models were selected using the following steps.

First, we divided each dataset for *S* and *A* (with outliers removed) into seven subgroups by mouse identity. For each of the seven mice, histograms of *S* and *A* were found to be not quite normally distributed. We therefore analyzed the data both with and without log-transformations to the *S* and *A* coefficients to improve normality.

Second, linear regression models were constructed for each mouse using both the original and the log-transformed data. We tested whether *S* and *A* were univariately associated with θ_bp_ or ϕ_bp_. Specifically, we tested the null hypothesis that the regression coefficients for θ_bp_ and/or ϕ_bp_ were equal to zero. In accordance with standard procedures of forward selection model evaluation, if the *P*-value for the independent variable coefficients was less than or equal to 0.05, we rejected the null hypothesis for the lower order model and proceeded to evaluate a higher order model (square of θ_bp_ and/or square of ϕ_bp_). The hypothesis that the regression coefficients for these higher order terms were equal to zero was then tested. For each quadratic order coefficient in the model, if the *P*-value was greater than 0.05, the model remained first order. If the *P*-value was less than 0.05, then the second order model was selected. We also considered the AIC as an additional metric to avoid overfitting. The higher order model was selected only if the AIC for that model was more than two points lower than the lower order model, and the independent variable coefficients were significant. We did not find any model for whiskers from an individual mouse to have independent variables greater than second order.

Third, if both basepoint parameters (θ_bp_ and ϕ_bp_) were univariately associated to either first or second order with either the original data or log-transformed *A* or *S*, both parameters were included in a multivariable linear regression model for each individual mouse. The method of forward selection was again used to introduce the terms as independent variables. We tested the hypothesis that the regression coefficients for θ_bp_ or ϕ_bp_ were equal to zero. If the *P*-value for the independent variable coefficients was less than or equal to 0.05, a second order model (square of θ_bp_ and/or square of ϕ_bp_) was fit and we again tested the hypothesis that the regression coefficients for these higher order terms were equal to zero.

We then compared the best-fit simple or multivariable linear regression models across all seven mice, for the original data and log-transformed *A* or *S*. An overall model, based on data from all mice together, was fit only after considering the results of the models fit to each individual mouse. A parameter (i.e. θ_bp_, ϕ_bp_ or their squares) was included in the overall model only if it had first appeared as a significant independent variable in at least six of the seven individual mouse models. Each of these parameters was then retained or discarded in the overall model using the same forward selection methods described for the individual models. To avoid overfitting, the order of the combined model was not allowed to exceed the highest order of the six out of seven individual mouse models.

#### Angles of emergence and basepoint radius as functions of θ_bp_ and ϕ_bp_

Of the 467 whiskers obtained from the eight mice, 461 were of sufficient quality to use in a 3D analysis of basepoints (*r*_bp_ θ_bp_ ϕ_bp_) and emergence angles (θ_w_, ϕ_w_, ζ_w_). To identify outliers for each parameter, the mean and standard deviation were calculated for whiskers grouped by their row and column identity. As before, the value of a given parameter for a particular whisker was eliminated if it was greater than two standard deviations above or below the mean.

Out of the dataset of 461 whiskers, 21 outliers (4.56%) were removed for *r*_bp_, 14 outliers (3.04%) were removed for θ_bp_, 19 outliers (4.12%) were removed for ϕ_bp_, 19 outliers (4.12%) were removed for θ_w_, 16 outliers (3.47%) were removed for ϕ_w_ and 13 outliers (2.82%) were removed for ζ_w_. Thus, after outlier removal, 440 values remained for *r*_bp_, 447 values remained for θ_bp_, 442 values remained for ϕ_bp_ and θ_w_, 445 values remained for ϕ_w_, and 448 values remained for ζ_w_.

We next constructed models for *r*_bp_ θ_w_, ϕ_w_ and ζ_w_ as functions of θ_bp_ and ϕ_bp_. To avoid overfitting, the best fit model for the parameters *r*_bp_, θ_w_, ϕ_w_ and ζ_w_ was found for each of the eight mice individually. We first determined that the parameters *r*_bp_, θ_w_, ϕ_w_ and ζ_w_ were all normally distributed for each of the eight mice. Therefore, a log transformation was not necessary and linear regression models were then constructed for each mouse as follows.

First, for each mouse, we tested whether r_bp_, θ_w_, ϕ_w_, and ζ_w_ were univariately associated with θ_bp_ and/or ϕ_bp_ using the forward selection procedure described previously. We did not find any models for whiskers from an individual mouse to have statistically significant independent variables greater than second order.

Second, if both basepoint parameters (θ_bp_ and ϕ_bp_) were univariately associated to either first or second order with *r*_bp_, θ_w_, ϕ_w_ and ζ_w_, then both parameters were included in a multivariable linear regression model for each individual mouse. Again, independent variables were added to the models using the method of ‘forward selection’. We tested the hypothesis that the regression coefficients for θ_bp_ and/or ϕ_bp_ were equal to zero. If the *P*-value for the independent variable coefficients was less than or equal to 0.05, a second order model (square of θ_bp_ and/or square of ϕ_bp_) was fit and we retested the hypothesis that the regression coefficients for these higher order terms was equal to zero. We also considered the AIC to avoid overfitting. The higher order model was selected only if both the independent variable coefficients were significant and the AIC for the higher order model was more than two points lower than the lower order model.

Finally, for each parameter (*r*_bp_, θ_w_, ϕ_w_ and ζ_w_), the best fit univariate or multivariable function of θ_bp_ and/or ϕ_bp_ was compared across all eight mice. An overall model, based on data from all mice together, was fitted only after taking into account the results of the models fitted to each individual mouse. This overall model was fitted similarly to the individual models, except that a parameter was included in the final combined model only if it had first appeared as a significant independent variable in at least six of the eight individual mouse models. If a parameter met this criterion it was initially included in the model, and retained or discarded using the same forward selection methods described for the individual models.

#### Error assessment

The Microscribe™ origin was defined by making a small divot in a piece of laboratory labeling tape on the operating table near the animal. At the start of each experiment, we calibrated the Microscribe™ by sampling this origin from five different orientations. The mean resolution over all scans and experiments was 0.5 mm. This error estimate includes any slight eccentricities of the tip, any deviation due to hand tremor of the user and the intrinsic precision limitations of the device. Note also that the 0.5 mm error does not compound along the arclength of the whisker because each point along the whisker was sampled independently.

As an additional estimate of measurement error, we performed simulations in which we randomly varied all points along the whisker arclength (including the basepoint) by ±0.5 mm in all three spatial dimensions. We then observed the effect on the 3D position and orientation of the whiskers. We performed these simulations for one rostral and one caudal whisker from each row, for a total of 10 whiskers. The maximum angular deviation of the whisker basepoint location was 0.015 mm in *r*_bp_, 1.07 deg in θ_bp_ and 0.53 deg in ϕ_bp_, and the maximum angular deviation of the whisker emergence angle was found to be 5.9 deg in θ_w_, 4.1 deg in ϕ_w_ and 28.7 deg in ζ_w_. The maximum error for ζ_w_ was a true outlier, as the next largest error among the remaining nine whiskers checked was 0.74 deg.

### Digitization of internal skull features

To position the skull and facial features in standard orientation, the (*x*, *y*, *z*) coordinates of the skull and face collected using the Microscribe™ were imported into MATLAB™. These coordinates were then rotated and translated to match the axis conventions shown in Fig. 1.

The data used for the external auditory meatus and lateral canal coordinates came from serial computed tomography (CT) scans of *Mus musculus* skull, specimen TMM M-3196, available through the digital morphology database DigiMorph.org (Demarest, 2001). The specimen was originally scanned along the coronal axis, for a total of 480 slices. Each 1024×1024 pixel slice is 0.02961 mm thick, with 0.02961 mm interslice spacing and a field of reconstruction of 28 mm. These images result in a resolution of 0.02734 mm in *x* and *y* positions (within each coronal plane slice) and an interslice resolution of 0.02961 mm in the *z* position.

We used Reconstruct™ to record the 3D coordinates of the bony labyrinth and other skull features in serial coronal CT images. Structures traced included the entire left and right bony labyrinths, the left and right external auditory meatuses, the lambdoid, sagittal and coronal sutures, and the lateral corners of the left and right upper incisors. Data were imported into MATLAB™ and we manually identified the (*x*, *y*, *z*) coordinates of the following skull features: (1) bregma and lambda; (2) the locations at which each of the two lateral canals terminated in a crista; (3) the lateral-most point of each of the two lateral canals; (4) five distinct points around the circumference of each auditory meatus; (5) the lateral corners of the incisors.

Points from the CT scan data were brought into the same reference frame as the Microscribe™ data by aligning a subset of corresponding points between the two datasets. These points included bregma, lambda and the corners of the incisors. We found the translations and rotations that brought these points into register and then applied these same translations and rotations to the points for all other features.

## RESULTS

The first two sections here describe the morphology of the mouse vibrissal array using linear regression, while the last four sections compare the morphology of the mouse and rat. In all equations, angles are measured in degrees, lengths in mm and curvature in mm^−1^. The model of the mouse is available at https://doi.org/10.5281/zenodo.7992354.

### Two-dimensional shape: arclength and curvature coefficient

The most basic description of two-dimensional (2D) whisker geometry includes arclength (*S*) and the intrinsic curvature coefficient (*A*). We quantified these two parameters as functions of the basepoint coordinates θ_bp_ and ϕ_bp_.

Arclength was best described as an exponential function of θ_bp_:
(5)

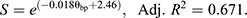


To provide a more intuitive sense for how whisker arclength varies across the array, Eqn 5 can be rewritten:
(6)

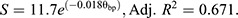


**Fig. 3.**
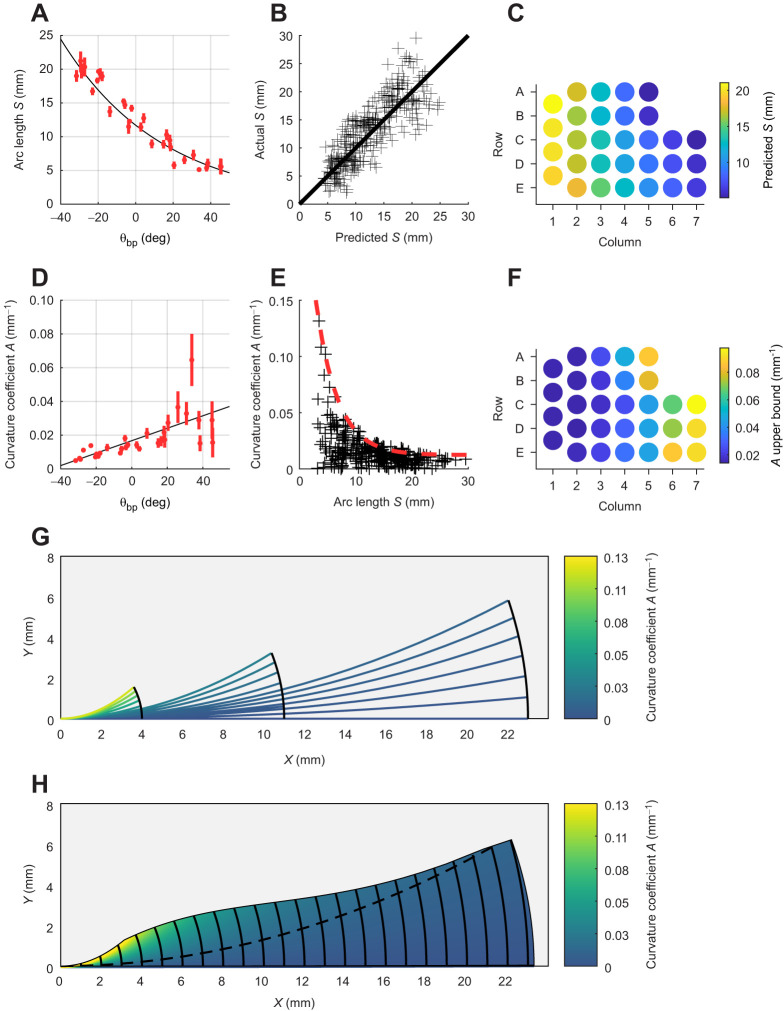
**Relationship between 2D whisker geometry and basepoint coordinates for the mouse.** (A) Whisker arclength (*S*) (*N*=343) can be described as a caudal–rostral decaying exponential function of the horizontal basepoint coordinate θ_bp_. Means±s.e.m. when grouped by whisker identity are in red. Black line represents Eqns 5 and 6. (B) Quality of fit for Eqns 5 and 6 is shown by plotting actual versus predicted values for *S*. (C) Predicted values for *S* across the array. (D) The curvature coefficient *A* (*N*=312) can be described as a linearly increasing function of θ_bp_ from caudal to rostral, but the quality of the fit is low because rostral whiskers have highly variable curvature. Means±s.e.m. when grouped by whisker identity are shown in red; black line represents Eqn 7. (E) The coefficient *A* is plotted as a function of *S*, highlighting that shorter whiskers have more variable curvature. Upper bound is given by Eqn 8 (red dashed line). (F) Predicted upper bound in curvature (Eqn 8) across the array. (G) A set of mock whiskers was generated to explore variability in the curvature coefficient. Each of the three black arcs connects whisker tips with a particular value of *S* and a range of values of *A*. The color of each whisker indicates its *A*-value. A small movement along a black arc (change in tip location) of a long whisker corresponds to a small color change (*A*-value), whereas a small change in tip location of a short whisker causes a large color change (*A*-value). (H) The analysis of G is shown for a continuous distribution of mock whiskers. Gray region indicates areas in which no part of the idealized whisker is predicted to lie. Dashed line indicates the boundary established by the *y*-intercept of Eqn 8. Above the boundary, *A* is larger than the intercept, so values of *S* can be computed from the equation. Below the boundary, *A* is smaller than the intercept, so values of *S* are fixed at their longest observed value.

Eqns 5 and 6 indicate that *S* decays exponentially from caudal to rostral (Fig. 3A). Whisker arclength is ∼11.7 mm near the rostro-caudal center of the array, where θ_bp_=0, and changes exponentially from the center. Experimental values for *S* vary between ∼2 and ∼30 mm. The quality of the fit for Eqns 5 and 6 is shown in the plot of actual versus predicted values for *S* (Fig. 3B), and Fig. 3C provides visual intuition for variations in *S* across the array.

As described in the Materials and Methods, whisker curvature was quantified using the 2D parameterization *y=Ax^2^*, fitted to the proximal 65% of the whisker. The curvature coefficient, *A*, was best described as a linear function of θ_bp,_ a relationship shown in Fig. 3D:
(7)

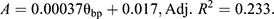


The *R*^2^ for Eqn 7 is low, due to the high variability in *A* for the rostral whiskers. However, Eqns 5–7 depend only on θ_bp_, indicating that mouse 2D whisker geometry is relatively consistent within a column.

Fig. 3E illustrates the relationship between curvature coefficient and arclength. Although curvature is particularly variable for short whiskers, a strong upper bound characterizes the relationship between *A* and *S*. An equation for the upper bound (Eqn 6) was generated by fitting to a series of maximum points using a moving average with a 4 mm window; this equation is visualized in Fig. 3F
(8)

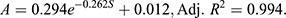


The results presented in Fig. 3D–F are subject to an important caveat. Although these panels show that the curvature coefficient is more variable for shorter whiskers, they do not imply that shorter whiskers have a more unpredictable tip location than longer whiskers. This statement is explained in the analysis of Fig. 3G–H.

To create Fig. 3G–H, we first identified the full range of *A* values found experimentally. We then solved for *S* using Eqn 8 for each value of *A* larger than the *y*-intercept (0.012). When *A* is smaller than the *y*-intercept, *S* is undefined and for these whiskers, *S* was fixed at the longest observed value. We then used these values for *A* and *S* to generate a set of ‘mock’, idealized whiskers that followed the curve *y*=*Ax*^2^ in standard Cartesian coordinates, where the whisker base was placed at the origin and its initial slope was aligned with the *x*-axis. These whiskers are idealized because their curvature is computed based only on their proximal 65% and then extrapolated to the tip. This approach accounts for the tendency of whisker tips to lie out of the primary plane of intrinsic curvature (Knutsen et al., 2008; Luo and Hartmann, 2023; Towal et al., 2011). A previous study provides an analysis of curvature that includes the full whisker length (Luo and Hartmann, 2023). Eighteen example mock whiskers (six whiskers for each of three different values of *S*), are shown in Fig. 3G. The color of a whisker indicates its *A* value. Each thick black arc shows the range of possible tip locations for a whisker with that arclength, as the parameter *A* is varied across its observed values. The top of each black arc terminates when a whisker of arclength *S* reaches its maximal observed curvature. The six shorter whiskers have more variable colors than the longer whiskers because shorter whiskers have more variable curvatures.

The analysis shown in Fig. 3G is extended across a more continuous distribution of whiskers in Fig. 3H. In this figure, each mock whisker still has a single color (representing its value of *A*), but whiskers are plotted densely enough to form a continuous color field. The small, colorful left corner indicates that curvature is more variable for shorter whiskers, but that this variability does not cause large tip displacements. In contrast, the right side is mostly dark blue, indicating that changes in tip locations of the longer whiskers are not associated with large differences in *A.*

The gray regions of Fig. 3H are those in which no whiskers are predicted to lie. This region is found by smoothly connecting the tops of the black arcs, bounding the curvatures for whiskers of given arclengths. However, note that, because *A* is calculated based only on the proximal 65% of the whisker, some of the actual whisker tips we sampled (31 of 312) would lie within the gray region. Given that the whiskers' distal tips are often damaged, the shape of any given mouse whisker tip will be variable and unpredictable. Therefore, an advantage of the present model is that simulated whiskers more closely reflect a ‘typical’ whisker instead of a particular individual mouse whisker.

### Angles of emergence of the whiskers as a function of basepoint coordinates

**Fig. 4.**
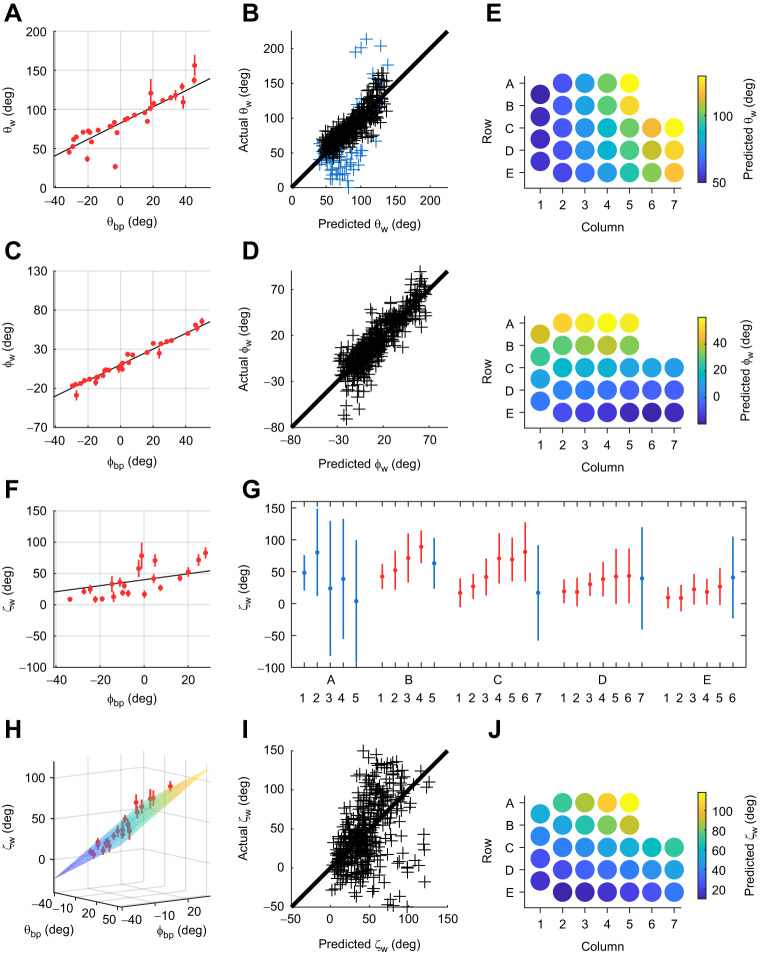
**Relationship between whisker angles of emergence and basepoint coordinates (θ_bp_ and ϕ_bp_) for the mouse.** (A) The horizontal emergence angle θ_w_ (*N*=445) increases linearly with horizontal basepoint coordinate θ_bp_ (*N*=447). Mean values±s.e.m. are in red; line of best fit (Eqn 9) is in black. (B) Fit quality for Eqn 9 with A-row whiskers identified in blue. (C) The elevation emergence angle ϕ_w_ (*N*=445) increases linearly with elevation basepoint coordinate ϕ_bp_ (*N*=442). Mean values±s.e.m. are in red; line of best fit (Eqn 11) is in black. (D) Fit quality for Eqn 11. (E) Predicted values for θ_w_ (top) and ϕ_bp_ (bottom). (F) Twist emergence angle ζ_w_ (*N*=448) is plotted against ϕ_bp_. Means±s.e.m. are in red and fit for Eqn 12 is in black. (G) Plotting ζ_w_ as a function of row (A–E) and column (1–6) reveals increases by column within rows B–E. Points and error bars show means±s.d. Data in blue were omitted from the second model; data in red were retained. (H) The fit improves when the A-row and the rostral-most column in each row are omitted, as shown by plotting ζ_w_ as a linear function of both θ_bp_ and ϕ_bp_. Means±s.e.m. are in red. The surface represents Eqn 13. (I) Quality of fit is reflected in the uniformity and dispersion of actual versus predicted values for F. (J) Predicted values of ζ_w_ across the array using Eqn 13.

As shown in Fig. 4A, the horizontal angle of emergence, θ_w_, was best described as a linear function of θ_bp_:
(9)

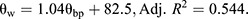


Although Eqn 9 represents the best relationship found while avoiding overfitting, the *R*^2^ value is relatively low. Moreover, plotting actual versus predicted values (Fig. 4B) reveals moderately high dispersion about the identity line, limiting the predictive value of Eqn 9.

However, values for θ_w_ were particularly variable for whiskers in the A row (Fig. 4B, blue points). After omitting these, the equation for θ_w_ is:
(10)

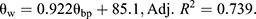
The elevation angle, ϕ_w_, was found to vary linearly only with ϕ_bp_:
(11)

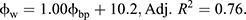


This relationship is shown in Fig. 4C, and Fig. 4D shows the quality of the fit by comparing predicted versus actual values.

The two colormaps of predicted θ_w_ and ϕ_w_ values across the array (Fig. 4E) confirm the visual intuition that more caudal whiskers emerge at more caudal angles than the rostral whiskers, while whiskers in more dorsal rows emerge at more elevated angles than whiskers in more ventral rows.

The angle ζ_w_, describes the twist of the whisker about its own axis. This angle can be computed accurately only if the whisker curves measurably away from its base. Therefore, whiskers shorter than 8 mm were excluded from the fits for ζ_w_. Even after omitting these whiskers, the best fit for the angle ζ_w_ was poor:
(12)

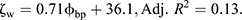


Fig. 4F shows values for ζ_w_ grouped by the row and column identity of the whisker and plotted as a function of ϕ_bp_, with Eqn 12 shown in black.

To explore the high variability in ζ_w_ we plotted it as a function of row and column (Fig. 4G), revealing that most variability comes from the A-row and the most rostral columns within each row. Models for each row individually were moderately to minimally predictive, as indicated by *R*^2^ values below 0.25. The *P*-value (>0.05) for the linear regression model for row A indicated that the values of ζ_w_ for these whiskers were not well-predicted by rostro-caudal position. In contrast, models for ζ_w_ for whiskers in rows B–E were well predicted by rostro-caudal position, as indicated by significant *F*-tests (*P*<0.005). These results suggest that a substantially improved model for ζ_w_ might be obtained after excluding the A-row and the most rostral whiskers. We therefore excluded these whiskers and re-fitted an overall model to the remaining 309 whiskers. As predicted, the fit improves significantly:
(13)

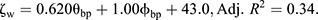


The reduced dataset, along with predicted values, is shown in Fig. 4H, and the quality of the model is illustrated in Fig. 4I. Predicted values from Eqn 13 are shown in the colormap in Fig. 4J. Our confidence in Eqn 13 is increased because its form and coefficients are similar to those for the rat (Belli et al., 2018):
(14)

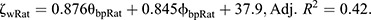


The rat and mouse models are not statistically different, as their 95% confidence intervals overlap for both predictors. It is also possible that a different choice for the horizontal plane would reveal a stronger relationship between ζ_w_ and θ_bp_ and/or ϕ_bp_.

### Comparisons between the mouse and rat

**Fig. 5.**
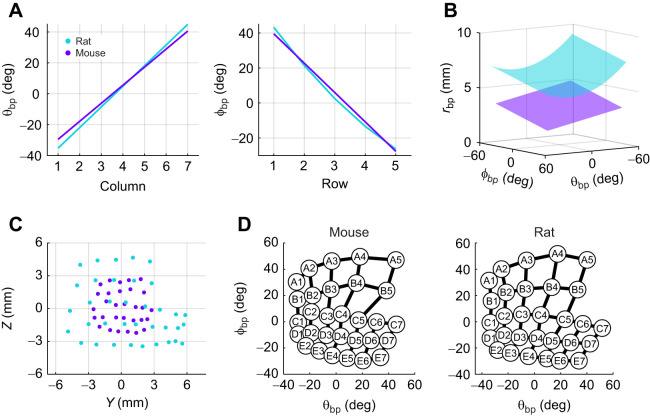
**Comparison of basepoint coordinates (*r*_bp,_ θ_bp_, ϕ_bp_) across species.** (A) The horizontal coordinate θ_bp_ (*N*=447) as a function of column, and the elevation coordinate ϕ_bp_ (*N*=442) as a function of row, are nearly identical for rat and mouse. Fit lines for each species intersect within the biologically relevant range of the predictor (row or column) and with similar slopes. (B) Plotting the radial coordinate *r*_bp_ (*N*=440) as a function of θ_bp_ and ϕ_bp_ highlights the head size difference between rats and mice. (C) Average whisker basepoint locations plotted in 3D in Cartesian coordinates. In A–C, data for mouse are purple and data for rat are cyan. (D) Average whisker basepoint locations in θ_bp_ and ϕ_bp_, for the mouse and rat. These panels implicitly normalize for *r*_bp_, revealing that the angular density is approximately the same between the two species, despite the size difference. Rat data are from Belli et al., 2018.

Table 1 compares equations for the mouse with equations for the rat obtained from Belli et al., 2018. Because the mouse and rat have the same number of whisker rows and columns, basepoint coordinates are easily compared. The first two lines of Table 1 and Fig. 5A show that rat and mouse arrays have similar relationships between the basepoint coordinates and row and column variables. One difference is that mouse ϕ_bp_ varies linearly with Row, while Belli et al., 2018 found that rat ϕ_bp_ varies quadratically with Row. However, when forced to a linear fit, the coefficients for the rat are not statistically different from those of the mouse, as indicated by overlap in the 95% confidence intervals of the two models (mouse interval [–16.28 to –17.29], rat interval [–16.81 to –17.92]). These models result in predicted values with an average difference between species of less than 2 deg.

**Table 1.**
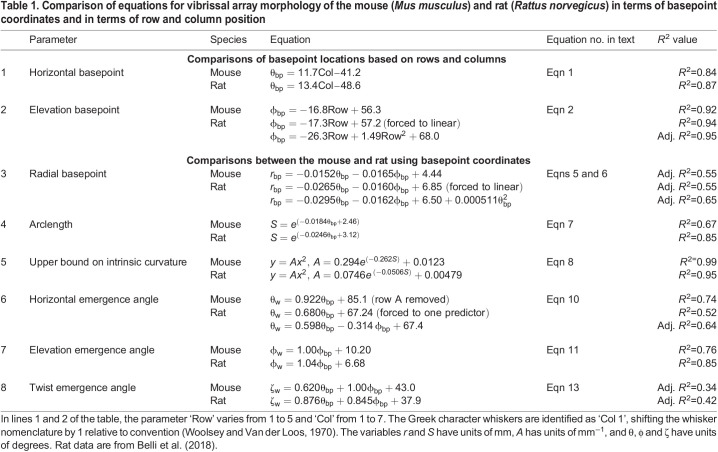
Comparison of equations for vibrissal array morphology of the mouse (*Mus musculus*) and rat (*Rattus norvegicus*) in terms of basepoint coordinates and in terms of row and column position

An important goal of the present work was to develop equations for whisker morphology and arrangement that can be compared across species, regardless of whether the species has an ‘orderly’ or ‘disorderly’ whisker array. Lines 3–8 of Table 1 compare the morphology of mouse and rat whisker arrays using θ_bp_ and ϕ_bp_ as independent variables. These types of comparisons could be performed for any whiskered animal; the basepoint coordinates can be measured independently of row and column position (lines 3–8 do not depend on line 1 and 2).

The parameter *r*_bp_ is compared in the third line of Table 1 and in Fig. 5B. Values for *r*_bp_ depend strongly on head size and are, on average, 56.1% larger in the rat compared with the mouse, with a range of 36.1% for the D5 whisker (the most similar) and 77.8% for the A1 whisker (the least similar). The larger *r*_bp_ values for rat were statistically significant with *P*<0.001 (independent samples *t*-test). This difference in size contrasts with the remarkable similarity between the two species for θ_bp_ and ϕ_bp_ by whisker identity (lines 1–2 of Table 1).

To obtain further intuition for the differences in scale across species, Fig. 5C shows the whisker basepoint locations for the two animals in the *y*–*z* plane. We found that, for the mouse, the total area occupied by whisker basepoints was 50.90±16.86 mm^2^, for a density of 0.66±0.44 whiskers mm^–2^, while the rat has a whisker area of 139.35±34.6 mm^2^, yielding a density of 0.24±0.05 whiskers mm^−2^. When normalized for the obvious size difference, however, mice and rats have approximately the same number of whiskers in the same angular region (Fig. 5D).

### Comparisons of 2D parameters (arclength, curvature, base diameter) in mice and rats

**Fig. 6.**
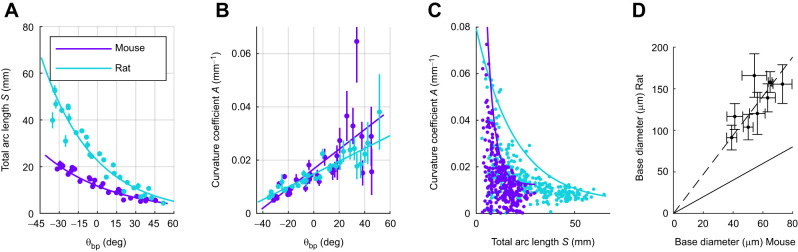
**Relationship between 2D whisker geometry and basepoint coordinates for mice and rats.** (A) Whisker arclength (*S*) can be described as a decaying exponential function of θ_bp_ from caudal to rostral. Curves represent best fit equations and dots and bars show whisker identity means±s.e.m. (B) Curvature coefficient (*A*) increases with θ_bp_ for both rat and mouse. Lines represent the best fit equations with dots and bars showing means±s.e.m. for whisker identity. (C) Scatterplots of *A* as a function of *S* for the mouse and rat reveal distinct upper bounds. (D) Comparison of base diameters of select mouse (*N*=28) and rat (*N*=146) whiskers. Error bars are standard deviations. Solid black line represents *y*=*x*. Dashed line represents the best fit line through the origin, *y*=2.35*x*. To ensure that the error bars are visible, *x* and *y* axes are not equal. Rat data are from Belli et al. (2018); mouse data are from Hires et al. (2016).

We compared the relationships that describe 2D whisker geometry for the mouse and rat (lines 4 and 5 of Table 1). Fig. 6A shows *S* as a function of θ_bp_, along with the fits given by equations in the fourth and fifth lines of Table 1. Whisker arclength has a larger range for the rat than mouse, reflected by the steeper exponential decay. The longest rat whisker was approximately twice as long as the longest mouse whisker (60 mm versus 30 mm).

Fig. 6B shows curvature coefficient (*A*) as a function of θ_bp_ for both animals, again highlighting increased variability for more rostral (shorter) whiskers. The coefficient does not differ significantly between the two species, as reflected in the overlapping confidence intervals. Fig. 6C plots the curvature coefficient as a function of arclength and the associated upper bounds, for mouse and rat. The upper bound on the rat is greater than that on the mouse for most whiskers, but there is considerable overlap.

To complete the analysis of 2D parameters, we used datasets from Hires et al., 2016 (mouse) and Belli et al., 2018 (rat) to compare the base diameters of selected mouse and rat whiskers (Fig. 6D). A total of 28 mouse whiskers and 146 rat whiskers were analyzed (see the Materials and Methods). On average, rat whiskers have base diameters ∼2.35 times larger than mouse whiskers.

### Angles of emergence of the whiskers as a function of basepoint coordinates in mice and rats

We next compared the 3D angles of emergence (θ_w_, ϕ_w_, ζ_w_) between the mouse and rat (Table 1, lines 6, 7 and 8.)

#### Horizontal angle

**Fig. 7.**
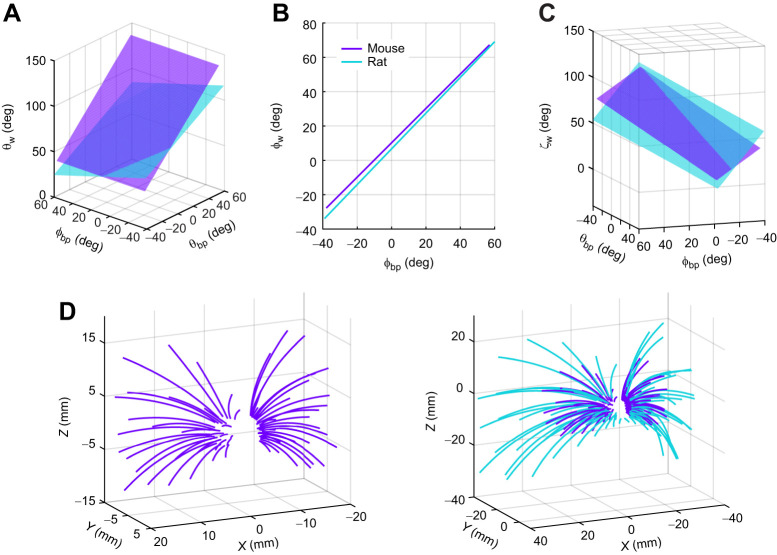
**Relationship between basepoint coordinates and angles of emergence for mice (purple in all panels) and rats (cyan in all panels).** (A) Mouse whiskers are oriented slightly more forward than those of rats, especially the rostral whiskers. (B) Mouse whiskers are ∼4 deg more elevated than rat whiskers. (C) The relationship between the twist angle ζ_w_ and basepoint locations shows that mouse and rat whiskers tend to be oriented concave downwards and forwards. (D) The full 3D model of the average mouse vibrissal array (left, purple) is similar to that of the rat (right, rat array overlaid in cyan). Rat data are from Belli et al. (2018). *r*_bp_: *N*=440; θ_bp_: *N*=447; ϕ_bp_: *N*=442; θ_w_: *N*=442; ϕ_w_: *N*=445; ζ_w_: *N*=448; S: *N*=343; A: *N*=312.

As shown in Fig. 7A, mouse whiskers tend to emerge from the cheek pointing more rostrally than those of the rat, especially the rostral whiskers. The mean value for θ_w_ in columns 6 and 7 of the mouse is 120 deg, compared with 97.0 deg in the rat. In contrast, θ_w_ is more similar between the mouse and rat for caudal whiskers: the mean value of θ_w_ for columns 1 and 2 (combined) is 59.3 deg for mouse and 51.0 deg for rat. Also, rat θ_w_ is a function of both θ_bp_ and ϕ_bp_, while mouse θ_w_ depends only on θ_bp_. These differences likely reflect the small size of mice, making it difficult to capture as much nuance in the model.

#### Elevation angle

Comparing ϕ_w_ between rats and mice (Fig. 7B) shows that mouse whiskers lie at slightly higher elevation than rat whiskers, as indicated by the almost parallel lines offset by ∼4 deg.

#### Twist angle

Although the relationship for ζ_w_ in the mouse is not strong (Eqn 13), it allows approximate comparison to the rat (Fig. 7C), with greatest differences found for the dorsal whiskers. In general, ζ_w_ is much more variable than the other angles for both species, in part due to limited measurement resolution. Using Eqns 1–13, the full 3D model of the average mouse vibrissal array is plotted in the left panel of Fig. 7D and compared with the average rat array (overlaid) in the right panel of Fig. 7D.

### Relationship of facial features to basepoint parameters (θ_bp_ and ϕ_bp_) for mice and rats

**Fig. 8.**
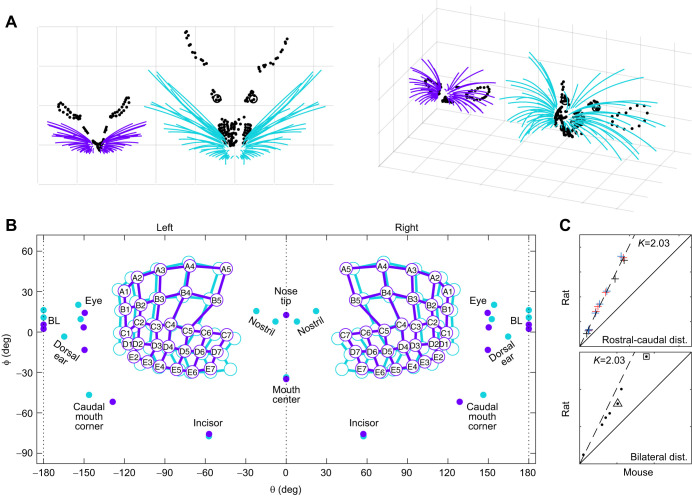
**Comparison between the facial features of the mouse and rat.** (A) Points on two individual animal's faces indicating the locations of eyes, ears, nose and mouth are shown with the whisker models for both rats and mice; all features are in the same coordinate system as for whiskers. Vertical and horizontal grid lines represent 20 mm. (B) Angular distances between facial and skull features for the mouse and rat. The origin is the centroid of all vibrissal basepoints. Bregma and lambda are marked ‘BL’. For both rat and mouse, bregma was slightly more elevated than lambda (recall that ‘horizontal’ is defined by the average whisker row plane). (C) Top panel: with the origin chosen as the centroid of all vibrissal basepoints, all rostral–caudal distances between facial features of the rat are nearly exactly 2.03 times larger than those of the mouse. Facial structures on the left and right are indicated by blue and red crosses, respectively. Bottom panel: most bilateral distances between facial structures of rat and mouse scale by a factor less than the value observed for rostral–caudal distances. The best fit line is given by *y*=1.49*x*+1.45. The triangle indicates the auditory meatus and the square indicates the ears. In both panels, the solid line represents *y*=*x* and the dashed line represents *y*=2.03*x*. Rat data are from Belli et al. (2018).

Finally, we quantified the locations of mouse facial and skull features relative to whisker basepoints. For visual intuition, Fig. 8A shows two views of the facial features of an individual mouse and rat. To compare relative facial feature locations despite differences in absolute scale, Fig. 8B superimposes these facial features in 2D angular coordinates. The origin is the centroid of all vibrissal basepoints, in the middle of the head. To gain physical intuition for this figure, the reader should imagine looking into the page with their nose at the origin, and then wrapping the figure around their head so that bregma and lambda are at the back. Only the width of the mouth appears to differ appreciably between the species.

These data allow us to compare both rostral–caudal and bilateral distances between facial features of the rat and mouse. The top panel of Fig. 8C shows that in the rostral–caudal dimension, all rat facial features are nearly exactly 2.03 times larger than those of the mouse. Bilateral distances between facial structures (Fig. 8C, bottom panel) are more variable and generally scale by less than the value of 2.03 observed for rostral–caudal distances.

**Fig. 9.**
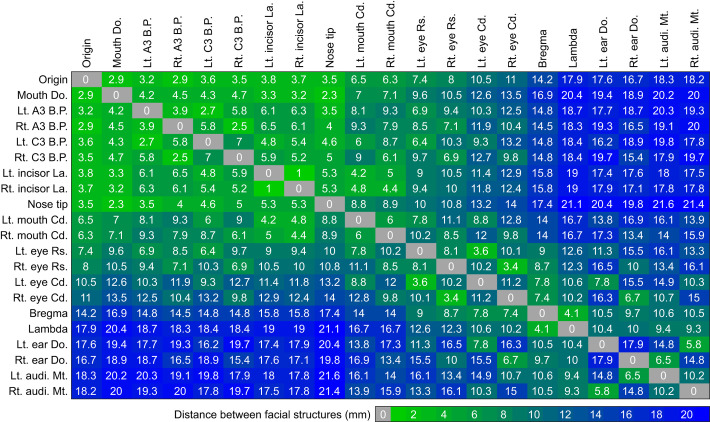
**Average absolute distance between facial and skull features and whisker basepoint parameters on the mouse.** Distance is in millimeters, ranging from ranging from zero (green) to 22.5 (blue). Abbreviations: Lt: left; Rt: right; Rs: rostral; Cd: caudal; Do: dorsal; La: lateral corner; B.P.: basepoint.

The straight-line distances between facial features of the mouse are shown in Fig. 9, highlighting clusters of rostral and caudal structures. In general, straight-line distances for mouse facial features are similar to those for the rat shown in fig. 9 of Belli et al. (2018). However, after normalizing for facial size, some important differences became evident. On each side of the mouse's face, the eye corners are slightly closer to the C3 whisker base points, and (again normalizing for facial size) the two ears of the mouse are further apart than the rat's, consistent with the strong bilateral differences in Fig. 8C.

## DISCUSSION

Because different animal species have different numbers and arrangements of whiskers (Muchlinski, 2010; Muchlinski et al., 2013), row and column position do not lend themselves to developing equations that can be used to compare whisker geometry across species. To overcome this problem, we developed an approach that uses 3D whisker basepoint positions to quantify whisker properties including arclength, curvature and emergence angles. Facial features can then be placed in this same coordinate system. Using this approach, we quantified and compared whisker and facial morphology across mice and rats.

### Variations in whisker arclength and intrinsic curvature

Consistent with previous studies on rats (Brecht et al., 1997; Pockock, 1914; Towal et al., 2011), the present work shows that whisker arclength increases exponentially from rostral to caudal across the mouse's face. Rat whiskers, which range in arclength from 10 to 60 mm, are approximately twice as long as mouse whiskers (Fig. 6A), in line with the ∼2× linear scaling of other facial features (Fig. 8). Data from previous studies (Belli et al., 2018; Hires et al., 2016) indicate that the base diameters of rat and mouse whiskers also differ by approximately a factor of two (Fig. 6D). The 2× linear scaling in both arclength and base diameter means that the volume (and hence mass) of rat whiskers is expected to be ∼8× greater than that of the mouse, comparable to the ∼10× body mass difference.

The intrinsic curvature of rat and mouse whiskers is described by a quadratic function, with a strict upper bound on maximum curvature and thus tip position (Fig. 3G and Fig. 6C). Rostral whiskers exhibit the largest variability in curvature coefficient, but that does not mean that their shapes are more variable. When a whisker is short, variations in its curvature coefficient have only a small effect on its tip position (Fig. 3G). Therefore, the coefficient of more rostral whiskers may not be under as strong selective pressure as the coefficient for longer whiskers.

### Whisker orientations at rest

The present work measured the locations of sensory structures in the anesthetized animal, so whisker emergence angles are the ‘resting’ angles. Both mice and rats move their whiskers through large angles during whisking behavior, with primary motion in the rostro–caudal direction. The horizontal angle θ_w_ can thus be considered an initialization parameter for simulations of whisking behavior, rather than a fixed parameter.

For both mice and rats the elevation angle φ_w_ is constant within a row (it varies only with φ_bp_) and the relationship between φ_w_ and φ_bp_ has one of the highest correlation coefficients (Table 1). We suggest that constant elevation angle (φ_w_) within each row simplifies the problem of localizing the height of an object in head-centered coordinates. Whisking behavior continuously changes the azimuthal angle (rostral–caudal) but the elevation angle (dorsal–ventral) remains relatively fixed. We predict that the single-variable linear relationship between φ_w_ and φ_bp_ is a characteristic feature of whisker-based tactile sensing that will generalize across all or most species.

In contrast to the single-variable relationship between φ_w_ and φ_bp_, the twist (ζ_w_) of the whisker about its own axis varies diagonally, and depends on both θ_bp_ and φ_bp_. In addition, the fit quality is poor, mostly because of measurement error. However, it is also possible that choosing a different head pitch to define the horizontal plane could reveal a stronger linear relationship. The diagonal orientation gradient could be particularly useful for determining the direction of a stimulus moving across the array. Stimuli such as air or water, which interact with many stationary whiskers simultaneously, will encounter different gradients of whisker twist depending on the animal's head pitch. These gradients could permit the animal to infer stimulus direction across a population of responsive neurons (Quist and Hartmann, 2012; Yu et al., 2016b).

### Reasons why facial and vibrissal morphologies of mice and rats should be similar, and why they should be different

There are several reasons to expect rats and mice to have similar facial and vibrissal morphologies. Though rats and mice diverged earlier than did humans and chimps [∼10 mya compared with ∼8 mya (Patterson et al., 2006) (Steppan et al., 2004)] they remain so physically similar that many non-experts confuse the two species. Both rats and mice are small, terrestrial, burrowing, cathemeral or crepuscular omnivores. Both species occupy a niche that relies on quick reproduction and on quantity rather than quality of offspring to maintain population (Berry and Bronson, 1992; Feng and Himsworth, 2013), although mice emphasize this strategy slightly more than rats (Berry and Bronson, 1992).

The two species also retain many less obvious adaptations in common, such as longitudinal chewing with interlocking molar cusps, probably inherited from an insectivorous ancestor (Lazzari et al., 2008). Most importantly for the question of whisker morphology, both rats and mice actively tap and brush their whiskers against objects during tactile exploratory behaviors. These motions, called ‘whisking’, help the animal determine object location, size, orientation and texture (Carvell and Simons, 1990; Grant et al., 2009; Knutsen et al., 2006; Krupa et al., 2001; Mehta et al., 2007; Pammer et al., 2013; Polley et al., 2005; Vincent, 1912). Facial muscles are also similar between the species, as is the total number of whiskers (Dörfl, 1982; Haidarliu et al., 2010).

However, there are some distinct differences between mice and rats that could produce differing selective pressures on facial and vibrissal morphology. Rats weigh about ten times more than mice, conferring a survival advantage given that small predators are less willing to take larger prey (Parsons et al., 2018). Although both species whisk, rats move their whiskers at average frequencies between 5 and 15 Hz while mice whisk faster, between 10 and 25 Hz (Jin et al., 2004; Welker, 1964)

Selective pressure for the morphological divergence of rats and mice could also emerge from differences in diet, as rats lack a gallbladder while mice retain theirs (McMaster, 1922), possibly assisting the mouse's relative emphasis on insectivory (Shiels et al., 2013). Although rats eat fewer insects, they are an occasional predator of mice (Yoshimura and Ueki, 1981). More evidence that the two species have specialized niches comes from their different colonization patterns. While the mouse spread across the Old World roughly following the expansion of human farming during the Bronze age (Auffray et al., 2008), the rat spread out of Asia much later, in the 18th century, after humans had already established urban environments.

Finally, the whisker systems of mice and rats also contain notable differences in the barrel cortex – barrels in rat cortex have ‘cell-dense’ centers compared with barrels with ‘cell-sparse’ centers in mice, a difference that arises in rats during postnatal development (Rice et al., 1985). Additionally, inter-barrel septa are much less prominent in mice compared with rats (Rice et al., 1985).

### Rat facial sensory structures may be ‘scaled-up’ versions of mouse facial sensory structures

The present results support the idea that the larger size of rats compared with mice is a derived (apomorphic) trait. After scaling, rats and mice have extremely similar whisker array and facial feature morphology (Figs 8, 9; Table 1). Both species exhibit strong linear relationships between θ_bp_ and column and between *r*_bp_ and ϕ_bp_. In addition, plotting whisker basepoint location as a function of ϕ_bp_ and θ_bp_, which normalizes whisker location to head size, reveals that mouse and rat whisker basepoints occupy strikingly similar positions. One difference between species is that the relationships between ϕ_bp_ and row, and between *r*_bp_ and θ_bp_, are linear for mice but quadratic for rats. These results indicate that mice and rats have snouts with similar mediolateral curvature but that the rat's snout curves slightly more dorsoventrally.

Thus, although the rat has a slightly more elongated face, consistent with the tendency for larger animals to have longer faces (Cardini and Polly, 2013), the most prominent difference between the two species is simply scale. These results also agree with findings that the molar-to-mandible ratio scales linearly between mice and rats (Cai et al., 2007). Notably, the bilateral (but not rostral–caudal) distance between the ears is disproportionately large for the mouse, perhaps helping to ensure larger interaural differences.

At the same time, other studies indicate that the common ancestor of mice and rats is likely to have been on the same scale as modern mice. For example, divergence in the mouse/rat lineage has been investigated using fossil molars from a probable common ancestor of mice and rats (*Antemus chinjinensis*), as well as later extinct murines (Kimura et al., 2015). These fossils indicate that *Antemus* molars are on the same scale as modern mouse molars. Given the essentially linear scaling between extant mice and rats found in the present work, we suggest that *Antemus* is likely to have had a similar facial feature arrangement, and that the facial morphology of the rat can be thought of as a ‘scaled-up’ version of the mouse.

### Utility of the model for simulations and neurophysiological experiments

Studying the nervous system using categorical stimulus sets (Bale et al., 2013; Jones et al., 2004; Lottem and Azouz, 2011; Shoykhet et al., 2000; Storchi et al., 2012; Stüttgen et al., 2008) can inadvertently mischaracterize the coding of primary sensory neurons (Bush et al., 2016a,b; Campagner et al., 2016; Severson et al., 2019). We anticipate that the present work will allow simulation of the multimodal sensory data that the mouse acquires during natural exploratory behaviors, similarly to other pose estimation or body dynamics software (Bolaños et al., 2021; Ramalingasetty et al., 2021; Zweifel et al., 2021). After adding multiple sources of variability and noise, these simulations can help establish rich, naturalistic stimulus datasets for use in neurophysiological experiments.
